# Effects of PM_2.5_ on People’s Emotion: A Case Study of Weibo (Chinese Twitter) in Beijing

**DOI:** 10.3390/ijerph18105422

**Published:** 2021-05-19

**Authors:** Siqing Shan, Xijie Ju, Yigang Wei, Zijin Wang

**Affiliations:** 1 School of Economics and Management, Beihang University, Beijing 100191, China; shansiqing@buaa.edu.cn (S.S.); weiyg@buaa.edu.cn (Y.W.); wangzijin@buaa.edu.cn (Z.W.); 2 Beijing Key Laboratory of Emergency Support Simulation Technologies for City Operation, Beijing 100191, China

**Keywords:** PM_2.5_, social media data, sentiment analysis, machine learning

## Abstract

PM_2.5_ not only harms physical health but also has negative impacts on the public’s wellbeing and cognitive and behavioral patterns. However, traditional air quality assessments may fail to provide comprehensive, real-time monitoring of air quality because of the sparse distribution of air quality monitoring stations. Overcoming some key limitations of traditional surface monitoring data, Web-based social media platforms, such as Twitter, Weibo, and Facebook, provide a promising tool and novel perspective for environmental monitoring, prediction, and evaluation. This study aims to investigate the relationship between PM_2.5_ levels and people’s emotional intensity by observing social media postings. This study defines the “emotional intensity” indicator, which is measured by the number of negative posts on Weibo, based on Weibo data related to haze from 2016 and 2017. This study estimates sentiment polarity using a recurrent neural networks model based on LSTM (Long Short-Term Memory) and verifies the correlation between high PM_2.5_ levels and negative posts on Weibo using a Pearson correlation coefficient and multiple linear regression model. This study makes the following observations: (1) Taking the two-year data as an example, this study recorded the significant influence of PM_2.5_ levels on netizens’ posting behavior. (2) Air quality, meteorological factors, the seasons, and other factors have a strong influence on netizens’ emotional intensity. (3) From a quantitative viewpoint, the level of PM_2.5_ varies by 1 unit, and the number of negative Weibo posts fluctuates by 1.0168 units. Thus, it can be concluded that netizens’ emotional intensity is significantly positively affected by levels of PM_2.5_. The high correlation between PM_2.5_ levels and emotional intensity and the sensitivity of social media data shows that social media data can be used to provide a new perspective on the assessment of air quality.

## 1. Introduction

With the advancement of industrialization in Chinese cities and the trend towards increasing urbanization in China, environmental issues are becoming increasingly pressing. Industrial pollution, automobile exhaust, the use of coal-fired heating, and other problems are resulting in a growing number of haze episodes [1]. According to the Bulletin on the State of China’s Ecological Environment in 2017, the days of heavy pollution in 338 cities of China account for 1.9% of the year; the days of severe pollution account for 0.6% of the year. The days with PM_2.5_ as the primary pollutant accounted for 74% of days with heavy or relatively severe pollution, days with PM_10_ as the primary pollutant accounted for 20%, and days with O_3_ as the primary pollutant accounted for 6%, therefore, PM_2.5_ is the main component of haze. Many studies have shown that severe haze affects not only physical and mental health, but also deals a heavy blow to society in many other ways. For example, haze can induce cardiopulmonary diseases [2,3]. Haze can stimulate or aggravate depression, which has a negative impact on people’s moods and social lives [4,5]. The increase in air humidity and pollution during haze episodes can cause damage to power equipment, which can seriously impact the safety of the power grid and the operation of power equipment [6,7]. In addition, the reduced visibility caused by haze can impact traffic safety, leading to the closure of aviation, highway, and railway lines, as well as traffic delays and congestion [8]. The effective identification and measurement of haze problems can help us to measure the severity of pollution quantitatively, improve people’s awareness of the need to avoid haze risks, and strengthen the determination of the government and individuals to reduce pollution.

**Figure 1:**
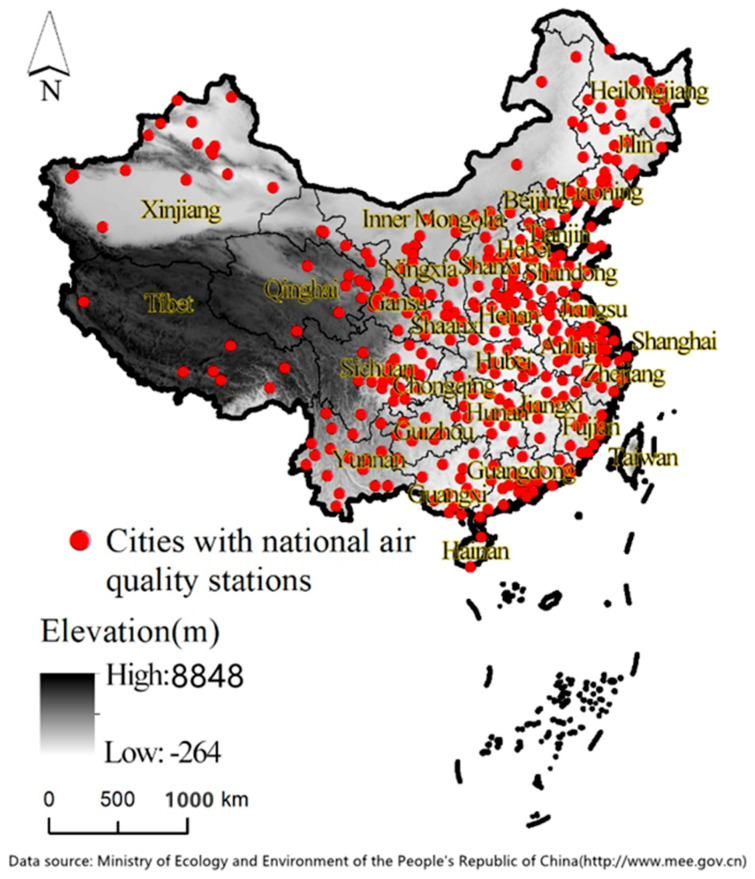
Distribution map of cities with national air monitoring sites (2018).

At present, air quality assessment continues to rely on statistical data from air quality monitoring stations. In general, the distance between the monitoring site and the site being measured impacts the accuracy of the air quality measurement. However, the coverage rate of air quality monitoring stations is still very low because of technical and economic constraints. Most air quality monitoring stations are located in economically developed areas in eastern China. Most areas in northeastern and western China are still not covered, especially Xinjiang, Tibet, Qinghai, and Inner Mongolia, as shown in Figure 1. According to the Ministry of Ecology and Environment of the People’s Republic of China (http://www.mee.gov.cn/ (accessed on 19 May 2021)), there are relatively few air quality monitoring stations, and most of them are in cities at the prefecture-level and above. In addition, limited and potentially non-representative monitoring locations in cities lead to an inability to accurately indicate air quality in unmonitored locations [9].

With the rise of social media, more people are using online social tools, such as Weibo and WeChat, to express their opinions and moods, thus creating a large amount of user-generated content. Among them, Weibo, as a social networking platform, had over 400 million monthly active users as of May 2018. Users from different backgrounds can share their opinions and views about various areas of social life on Weibo [10]. There are certain netizens who follow current events and express their views on a variety of issues, including immediate events, social marketing, and international affairs. As an issue of topical importance, haze has also attracted the attention of netizens. Every interaction on Weibo reflects netizens’ psychology. Posting comments and forwarding information shows people’s interest in a topic, and the content posted on Weibo reflects the views and emotional tendencies of Weibo users. Compared to using traditional data, using social media data has the following advantages [11]. (1) The data are relatively real and reliable, as they come directly from user-generated content. (2) The data volume is large. Regarding Weibo, with more than 400 million monthly active users, its data volume is close to EB magnitude (EB is a computer storage unit, and EB magnitude represents a great volume of data). The extensive data from Weibo cover a wide range of users and geographic regions. (3) There are many types of data. Structured report data and unstructured data, such as images and audio recordings, are available simultaneously. (4) The data are often instantaneously available. Users can upload data anytime and anywhere, which reduces the labor cost of traditional data collection and solves the problem of data lag. Of course, it is undeniable that the application of social media data presents many challenges. (1) The emergence of paid posters publishing positive posts on purpose can affect the data’s validity [12]. (2) Differences in user concentration by geographical location lead to uneven distribution of social media data according to the User Development Report by the Weibo Data Center in 2017. (3) Real-time social data may accelerate the spread of rumors because of a lack of information review mechanisms. (4) The large volume and real-time nature of the data impose higher requirements for the speed and abilities of data processing [11]. Many scholars have adopted data pre-processing methods, such as sampling and filtering, to avoid the weaknesses of using social media data as much as possible, and social media data are increasingly being used in different research areas [13,14,15]. Enterprises can take advantage of social media to establish target audiences and target marketing efforts based on social media data [10]. For example, historical influenza data are real but delayed, but data from Twitter provide a real-time assessment of influenza, and a combination of the two can improve accuracy in predicting influenza trends [16]. Social media data can also be used to monitor public opinions on the Internet, providing guidance for the government so that it can improve public perception and formulate policies [17].

In the Web 2.0 era, many studies have begun experimenting with air quality monitoring based on social media data. Although big data may have problems of noise and low-value density, social media data, through cleaning and selection, can compensate for the poor timeliness and low coverage of traditional data and effectively support various applications. For example, in terms of social economy, studies have validated the reactions of netizens to stock trends [18], and sentiments expressed on Weibo can be used to predict the performance of stocks [19] and to analysis -disaster loss [20,21]. In the realm of public affairs, some scholars have attempted to use Twitter data to monitor epidemic diseases in real-time and to study disease transmission routes through complex networks [16]. Social media can also be used to monitor disasters and assess disaster losses in real time [22]. In the political field, Tsou used observations from social media to successfully predict the outcome of the 2012 American presidential election [23]. In the field of air quality, the application of social media is still in its infancy and has great prospects. Wang found that the number of air pollution-related posts on Sina Weibo can reflect the true level of particle pollution [24], thus demonstrating that social media data can enrich existing air pollution monitoring data. On the basis of the Markov Random Field model, Mei used the number of daily Weibo posts to predict AQI (Air Quality Index, which is used to describe air quality conditions) [25]. Ni et al. combined social media data with meteorological factors and used a time series to predict short-term PM_2.5_ levels [26]. Li et al. found that environmental pollution can aggravate people’s feelings of loss and tension [27]. To date, studies of air quality based on social media data have relied on the frequency of netizens’ posts, but they have seldom considered the impact of air quality on netizens’ sentiments. In addition, researchers conducting studies on public sentiment have used questionnaire surveys that collect responses indicating people’s views and attitudes toward the events and measure their degree of satisfaction with the way the events are handled. This differs from direct analysis of public sentiment based on the content of social media posts, and insufficient sample sizes and ad hoc scoring methods may present difficulties for researchers.

Our study introduces the concept of emotional intensity by combining netizens’ moods and their frequency of posting. People’s emotional intensity is measured by the number of Weibo posts with a certain sentiment polarity that refers to emotional tendencies to feel something, such as positive and negative emotions, and the relationship between PM_2.5_ levels and emotional intensity is investigated by exploring the influence mechanism of PM_2.5_ levels on posting behavior. The study provides a new perspective on, and data source for, air monitoring by demonstrating a high correlation between PM_2.5_ levels and the emotional intensity of netizens. To achieve this objective, three specific issues are investigated: (1) the sentiment polarity in Weibo posts as estimated by the LSTM (Long-Short Term Memory) neural network model, (2) the strength of correlation between PM_2.5_ levels and emotional intensity as measured by the Pearson correlation coefficient, and (3) the impact of PM_2.5_ levels on emotional intensity as shown in a multiple linear regression model.

The innovative aspect of the study lies in the following factors. (1) On the basis of real-time social data, the impact of PM_2.5_ levels on the emotional intensity of netizens was explored. (2) The number and sentiment polarity of Weibo posts related to haze account for all the sentiments expressed by netizens, the number of negative Weibo posts per day was selected as the measurement indicator of emotional intensity, and the high correlation between the indicator and PM_2.5_ levels was verified through experiments.

## 2. Data and Method

**Figure 2:**
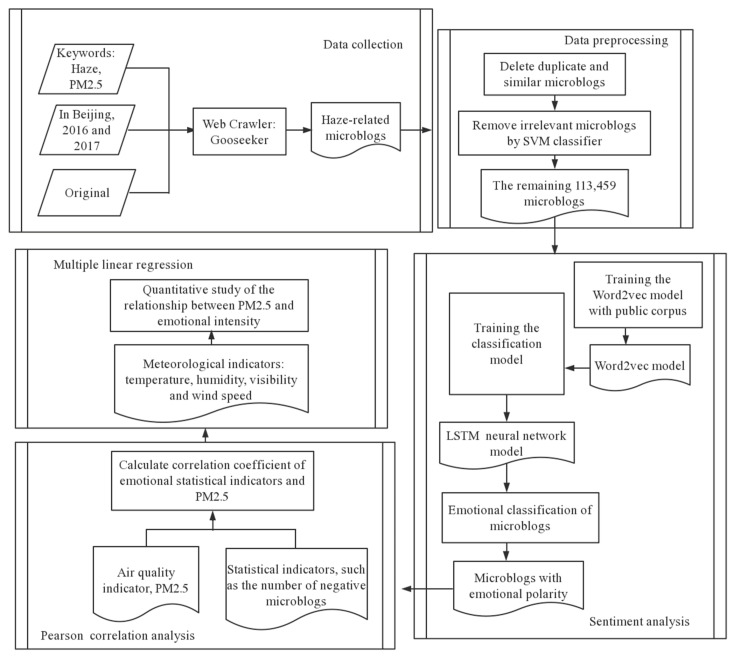
Analytical framework for using social media data for PM_2.5_ research.

Figure 2 shows the theoretical and methodological process of the study. (1) Data collection: Capture the original Weibo posts related to haze in 2016 and 2017 in Beijing through GooSeeker Web crawler software. (2) Data preprocessing: Delete duplicate crawling and similar Weibo posts, and filter Weibo posts unrelated to haze. (3) Sentiment analysis: Use the LSTM (Long-Short Term Memory) classifier to judge the sentiment polarity. (4) Pearson correlation analysis: Verify the relationship between the number of negative Weibo posts and high PM_2.5_ levels through the Pearson correlation coefficient. (5) Linear regression analysis: Build a multiple linear regression model and conduct a quantitative study of the degree of correlation between the number of negative Weibo posts and high PM_2.5_ levels, which further validates the correlation between the two.

### 2.1. Data

Considering that PM_2.5_ is the main component of haze, this paper uses PM_2.5_ levels as a monitoring indicator of air quality. This study obtained real-time data from air quality monitoring stations in urban areas of Beijing in 2016 and 2017 from the China National Environmental Monitoring Centre website (http://www.cnemc.cn (accessed on 19 May 2021)). Considering the mobility of air and the travel of Weibo users between cities, such as from Haidian District to Chaoyang District in Beijing, we took the average hourly PM_2.5_ data of each monitoring station to obtain the daily PM_2.5_ concentration in Beijing. The temperature, humidity, visibility and wind speed data used in this paper came from the Wunderground platform (https://www.wunderground.com/ (accessed on 19 May 2021)) as of 2016 and 2017. The data from this website are considered highly reliable, and they have been widely used by environmental research scholars.

The Weibo data were obtained from the API (Application Programming Interface) of Sina Weibo using Gooseeker data crawler software. Because the investigation’s focus is related to haze, and also to avoid the excessive workload of crawling all of the network data, we used “haze” and “PM_2.5_” as keywords, and we set the crawler only to capture “original” Weibo posts containing these keywords. This study assumes that the users who post such things on Weibo are more concerned about environmental problems, such as haze, than other netizens. In the selection of data, we did not consider reposted or forwarded messages because this study relies on the real-time nature of social media data and non-original microblogs often go through a long process of transmission so that they cannot reflect the dynamics of PM_2.5_ levels in the real world. The time was set to 2016 and 2017 in the “advanced search” settings of Weibo, and the “location,” which refers to the address of the user who posts on Weibo, was set as Beijing. The traditional method, of setting the “registered address” as Beijing, cannot solve the sample noise problem of the actual location of the Weibo user being located somewhere other than Beijing because of population mobility. In contrast, the data crawling method is more reasonable, and it helps to better track the relationship between the general sentiment of people in Beijing and air quality. This study collected a total of 203,882 Weibo posts through this method.

Because of the problem of repeated crawling and haze-related advertisements, this study preprocessed the crawled data, removed duplicate and similar Weibo posts, and filtered out useless information. Duplicate Weibo posts mainly come from the redundant work of crawlers, and we judged whether any Weibo posts were repeated in the dataset using Python. If there were duplicated posts, only one of them was preserved. We also found some semantically similar Weibo posts that were mostly posted by fans of stars and advertising information. We filtered them out by calculating the Levenshtein distance between Weibo posts. For other official Weibo posts of air quality reports without subjective emotion and advertisements for products that were mostly used for promotion of anti-haze products, this research manually labeled some Weibo posts, trained an SVM (Support Vector Machine) model to classify some posts as irrelevant and eliminated useless information. The classification accuracy of SVM models can reach 93%. A total of 113,459 messages were preserved. The top 10 most popular words in all of these posts are haze, Beijing, weather, serious, mask, air, feeling, blue sky, mood, and life, centered on the themes of “Beijing,” “weather,” “protective measures,” and “mood.” The words also reflect netizens’ concern about the haze and the impact of haze on peoples’ mood.

### 2.2. Research Method

#### 2.2.1. SVM Classification Model

An SVM (Support Vector Machine) is a machine learning method based on statistical learning theory. To find an optimal compromise between the complexity of the model and learning ability, it can map the sample spaces into a high-dimensional feature space through kernel functions, so that the nonlinear separable problems in the original sample spaces are transformed into linearly separable problems in the feature space [28,29]. The algorithm only needs to abstract a certain amount of text into vectorized training text data by calculation, which improves the accuracy rate of classification.

**Figure 3:**
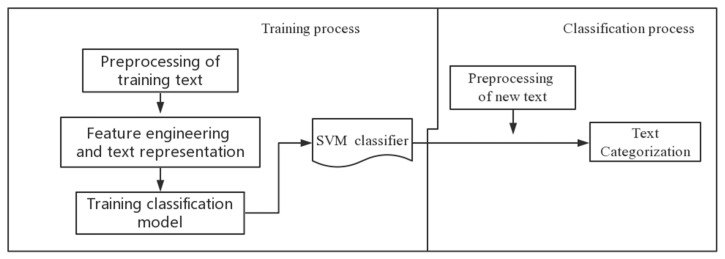
Implementation of the SVM classifier.

This study uses the SVM classification model to filter out useless Weibo posts. The specific steps are as follows: (1) use Chinese Jieba text segmentation tools to split Weibo posts; (2) use the TF-IDF (term frequency-inverse document frequency is a statistical method used to assess the importance of a word for a file set or one of the files in a corpus) to calculate the weight of the feature, select representative features from a large number of features, and complete the vectorized representation of the sample; (3) use the vectorization sample set to train the classification model, and test the accuracy; (4) use the trained model to classify the new text. This model is implemented on the basis of the system of SVM, as shown in Figure 3.

#### 2.2.2. Text Sentiment Analysis

Our study estimates sentiment polarity by a recurrent neural networks model based on LSTM (Long-Short Term Memory), which is an effective text sentiment classifier that can be used to process a massive amount of data [30].

First, to allow the computer to process natural language, the Weibo posts need to be modeled. In the process of modeling, problems such as the curse of dimensionality and weak generalization of models are prone to occur [24,31], and the Word2vec model presents a solution to these issues. Word2vec can vectorize each word quickly and efficiently according to a given corpus and map words with similar meanings to nearby positions in the vector space to achieve dimensionality reduction [32]. In the study, we used the 1.3 million-microblog corpus in the local database to train and obtain the Word2vec model.

Of the remaining 113,459 microblogs in the preprocessing stage, we sampled 1800. Then we manually judged the sentiment polarity of these data and labeled them as “positive” or “negative.” We arranged for three people to label a set of data together and then obtained the final label result by means of the mode, which guaranteed the accuracy of our data labels. Vectorization of the labeled data was performed with the trained Word2ve model.

**Figure 4:**
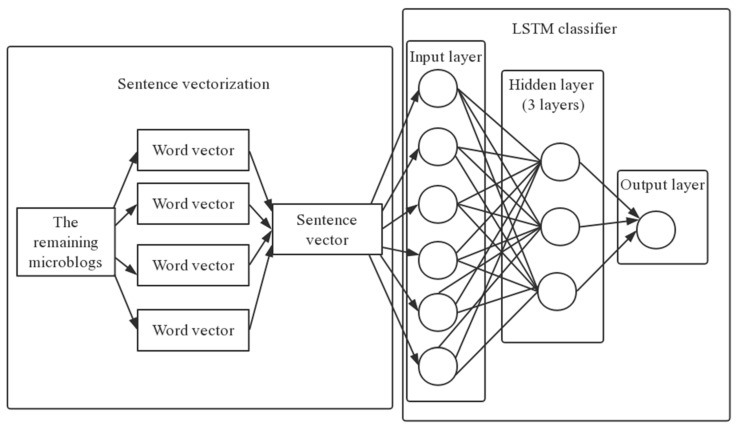
Diagram of the LSTM model.

We built a deep neural network based on LSTM (Long-Short Term Memory) for text sentiment classification, as shown in Figure 4. To fit data in this model better, this study implemented linear stacking of multiple network layers through sequential models [33]. The multilayer neural network included the input layer, hidden layer, and output layer [34]. Then we divided the 1800 vectorized and labeled data into a training set and a test set according to the principle of 8 to 2; that is, there were 1440 data for training of the classification model and 360 data for verifying the classification effect of the model. Finally, the classification accuracy of the trained model reached 67.8% in the test set. We used the trained classification model for the 113,459 Weibo posts to be classified and found the probability that each Weibo would be a negative Weibo post. We set the emotional threshold to 0.5, meaning that a probability greater than or equal to 0.5 indicated Weibo posts with a positive mood, while a probability under 0.5 indicated Weibo posts with a negative mood.

#### 2.2.3. Pearson Correlation Analysis and Indicator Selection

The Pearson correlation coefficient can be used to measure the relationship between two variables, X and Y, with values between −1 and 1. A value approaching 0 indicates no correlation, and a value approaching 1 or −1 indicates a strong correlation.

Some scholars have conducted relevant empirical studies to assess the impact of air pollution on people’s subjective feelings and behavior. Throughout the existing studies, the selected explained variables include people’s behavior and subjective feelings, expressed as happiness and satisfaction with life. The selected explanatory variables can be divided into six categories: air pollution, meteorological factors, other environmental variables, demographic variables, income, and social factors. Among them, air pollution has a negative impact on people’s subjective feelings and behavior [35]. Among meteorological factors, a certain amount of increase in temperature can have a positive impact on people’s emotions [36]. However, there is an inverted U relationship between temperature and emotion; the continuous rise of temperature adversely affects the mental state of people [37,38]. Emotional fluctuations have a significant relationship with sunshine duration. With the sun exposure time gradually shortening in the fall and winter, people’s brain activity and actions will change accordingly, and their negative mood cannot be effectively relieved, leading to the accumulation of a negative mood [39,40]. The indicators “wind speed,” “clouds,” “rain,” and “snow” have a negative impact on people’s emotions and behavior [41]. It is difficult for people to be in a good mood when the weather is bad. Among demographic variables, the better people’s self-development is, the easier it is to stay in a good mood; hence, the indicators “education level,” “employed,” and “health status” are positively related to people’s emotions and behavior [40]. Older people have a better mentality [42], and women are more optimistic about life [38]. People face many challenges after forming a family, and therefore the indicators “Married” and “Family size” have a negative impact on people’s emotions [36,40]. Income has a positive impact on people’s emotions [35]. People are also happier on weekends and holidays [38,41].

Combining the available data, we select a relevant set of determining factors of people’s emotions including the level of PM_2.5_, temperature, humidity, wind speed, precipitation, sea level pressure, holidays, weekends, and major events. In the study, the number of negative Weibo posts and the share of negative Weibo posts were regarded as a measure of emotional intensity. We control for temperature, humidity, wind speed, precipitation, sea level pressure, and other factors in the study. On holidays and weekends, people’s negative mood may be effectively relieved [43], so we add holidays and weekends to the control variables. Considering the impact of major events on people’s emotions, we combine the Weibo text to query the major events of 2016 and 2017 and screen the two major events of the Rio Olympic Games and the 19th National Congress of the Communist Party of China. At the 19th National Congress, information on the 13th Five-Year Plan, which is related to the social and economic development of the country over the next five years, the happiness of the people, and the prosperity of the country, was announced. Therefore, this had a positive effect on the public’s mood. A dummy variable indicating whether a major event occurred on a given day is added to the model. In addition, we believe that the seasons affect people’s emotions, and so our study controls for seasonal fixed effects.

#### 2.2.4. Multiple Linear Regression Model

A phenomenon is often associated with multiple factors. So, it is more effective and more realistic to predict or estimate the dependent variable using a combination of multiple independent variables instead of only one independent variable. In order to further verify the influence of PM_2.5_ levels on the emotional intensity of the public, we decided to construct a multiple linear regression model as shown in Equation (1), and we make the following hypothesis.

Hypothesis: There is a positive correlation between PM_2.5_ levels and people’s emotional intensity.

EI_t_ = β_0_ + β_1_PM_2.5t_ + β_2_X_t_ + β_3_Holiday_t_ + β_4_Weekend_t_ + β_5_Event_t_ + δ_t_ +ε_t_ (1)

EI_t_ represents the explained variable, that is, the emotional intensity. PM_2.5t_ represents the explanatory variable, that is, the air quality indicator, which is the core variable of concern. According to our hypothesis, the regression coefficient of this variable should be positive because, the higher the concentration of PM_2.5_, the stronger the tendency of people to make negative Weibo posts; thus, levels of PM_2.5_ are positively correlated with people’s emotional intensity. X_t_ represents a series of meteorological variables, including temperature, humidity, wind speed, precipitation, and sea-level pressure. The model includes the dummy variables of Holiday_t_ and Weekend_t_, which reflect whether a day is a legal holiday or a weekend. A dummy variable Event_t_, indicating whether a major event occurred on the day, is also added to the model. The study also controls for the seasonal fixed effect δ_t_. ε_t_ is a random error.

## 3. Results and Discussion

Each Weibo post is marked with an emotional label through the LSTM classifier. By counting the daily number of positive and negative Weibo posts, and determining the proportion of positive and negative Weibo posts to the total number of Weibo posts, we found that 74,613 Weibo posts among the 113,459 observed had a positive mood and 38,846 Weibo posts had a negative mood. The results show that, although the social network is full of negative posts that reflect resentment and dissatisfaction, positive posts are still the norm.

### 3.1. The Results of Pearson Correlation Analysis

The results of the Pearson correlation analysis are shown in Table 1. The number of positive and negative Weibo posts, the total number of daily Weibo posts, and the proportion of positive and negative Weibo posts are all significantly related to PM_2.5_ levels, with a statistical significance of less than 0.01. This study also observed an interesting phenomenon: the numbers of positive and negative Weibo posts are positively correlated with PM_2.5_ levels. The reasons for this result are as follows. (1) Sentiment polarity shows people’s dominant emotions as expressed in Weibo posts, rather than simply the blogger’s opinion of haze. Take this crawled Weibo post, for example: “The haze these days is killing me. Today, let us take our cameras and find a blue sky and green trees.” The first half of the sentence explains the adverse effects of haze on the body and mind, showing the blogger’s disgust with the haze, but in the latter half, the blogger decided to go outside, demonstrating a very positive attitude. Taken as a whole, we believe that the dominant emotion of this post is positive. People’s mood is affected by stimulating, physiological, and cognitive factors [44]. As one of the stimulating factors, haze may induce negative emotion, but emotion is also affected by other factors. In general, there are both negative and positive Weibo posts related to haze. (2) The more serious the haze, the more likely it is to attract people’s attention and cause netizens to make haze-related Weibo posts. There are also many haze-related Weibo posts with positive and negative moods. Therefore, both positive and negative Weibo posts are positively related to PM_2.5_ levels. Among them, the correlation coefficient between the number of negative Weibo posts and the PM_2.5_ level reaches 0.667, which is much higher than that between positive Weibo posts, or all Weibo posts, and the level of PM_2.5_: 0.589 and 0.625, respectively, which reflects the emotional classification. The effect is also consistent with our perception that the worse the air quality, the stronger the desire to make haze-related Weibo posts, and that, the more intensely the public’s mood is affected by haze, the greater the number of negative Weibo posts will be. The number of negative Weibo posts may not only reflect people’s attention to the haze, but also reflect the polarity of netizens. This study introduces the concept of emotional intensity.

**Figure 5:**
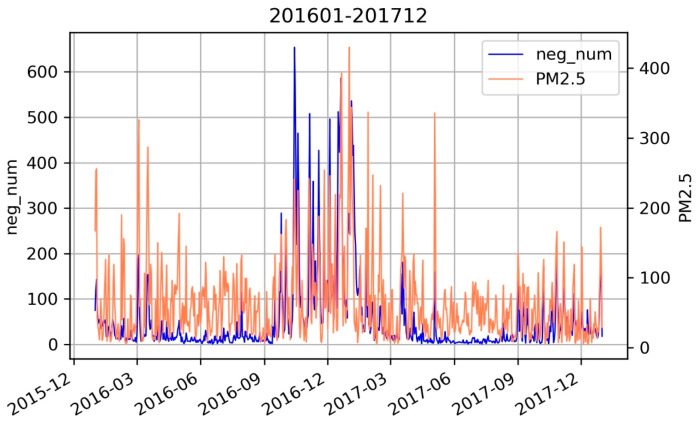
The number of negative microblogs and concentration levels of PM_2.5_.

This study has drawn a line graph of the emotional intensity indicator from 2016 to 2017: the number of negative Weibo posts and the air quality indicator PM_2.5_, as shown in Figure 5. It can be seen that the level of PM_2.5_ and the number of negative Weibo posts were high during the period from October 2016 to February 2017, indicating that the high incidence of haze in Beijing is concentrated in autumn and winter. This is related to reduced airflow and the use of coal-fired heating in autumn and winter [45]. Studies have shown that, in terms of the environmental cost of coal consumption, the problem of atmospheric pollution represented by haze is particularly serious. According to the China Climate Bulletin and the Energy Statistics Yearbook, the average annual haze duration in China before 2003 was less than 9 days. However, since 2004, it has grown rapidly, with an average annual value of 12 to 20 days today. In 2013, China’s average annual haze duration was as high as 36 days. The year 2013 was the peak year for China’s coal consumption, and it was also the year with the largest annual average haze duration in China. After 2014, as national coal consumption decreased, the national average haze duration began to decline. From Figure 5, it can be intuitively observed that the air quality in 2017 was significantly better than that in the winter of 2016, which is also inseparable from environmental supervision in Beijing. From the trend graph of the level of PM_2.5_ and the number of negative Weibo posts, we can see that the number of negative Weibo posts changes in the same direction as PM_2.5_, and the time of occurrence of extreme points on the two-fold lines is basically the same. The trends of the two are consistent. From this visual representation, we found that the level of PM_2.5_ is related to the number of negative Weibo posts.

**Figure 6:**
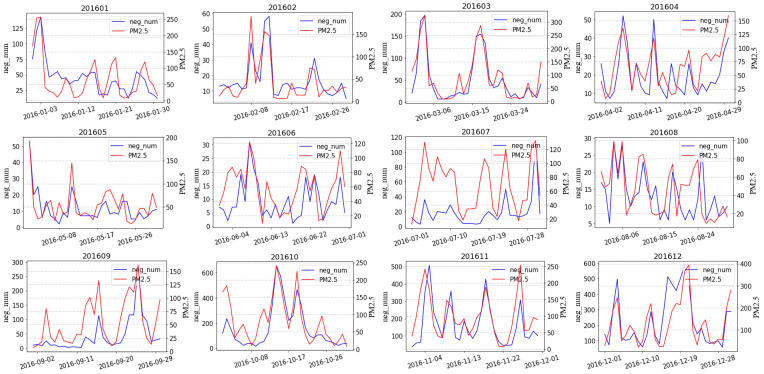

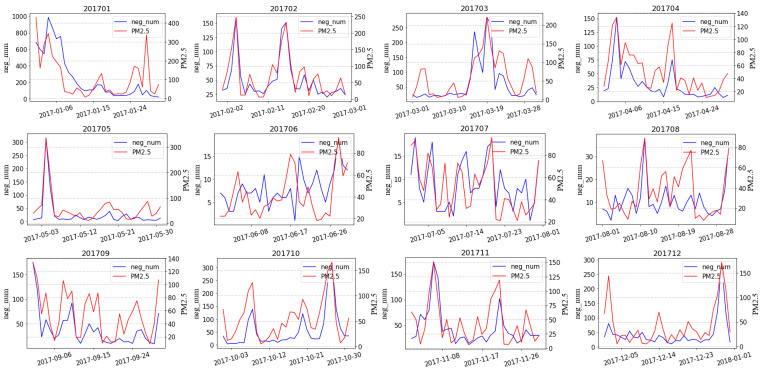
The trend of negative microblogs and concentration levels of PM_2.5_ for every month during 2016 and 2017.

**Figure 7:**
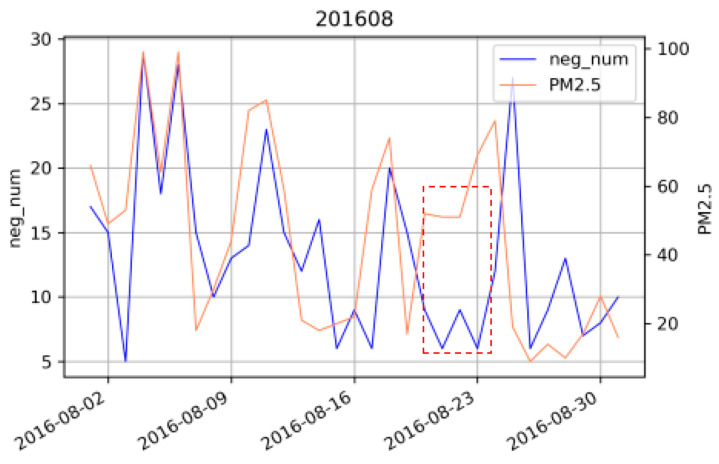
The trend of negative microblogs and concentration levels of PM_2.5_ in August 2016.

This study demonstrated a monthly Pearson correlation coefficient and significance between PM_2.5_ levels and negative Weibo posts in 2016 and 2017, as shown in Table 2. The data from all months passed a significance test. Figure 6 shows the line chart of PM_2.5_ levels and the number of negative Weibo posts in each month. This study found that the changing trend of PM_2.5_ levels and negative Weibo posts was basically the same, which preliminarily verified the idea that social media data can be used to infer air quality levels in real-time by monitoring the emotions of netizens. Combining the results presented in Table 2 and Figure 6, it can be observed that there is a large difference in the changing trend of the level of PM_2.5_ and the number of negative Weibo posts for some months. For example, the correlation coefficient in August 2016 was 0.461; in June 2017, it was 0.527; and in July 2017, it was 0.566. It can be observed that the major difference between air quality and the emotional intensity trend is concentrated in the summer. The weak correlation between the two may be due to better air quality and a smaller influence of the haze on the public’s mood. In addition, the mood is affected by multiple factors, and major events can also affect the mood of the whole network. This study takes August 2016 as an example, as shown in Figure 7, to analyze the reasons for the significant differences. It can be concluded that PM_2.5_ levels continued to rise during the period from August 19 to August 23, but the number of negative Weibo posts did not increase, remaining at a very low level. Considering that the mood of the whole network may be affected by other significant events, this study checked the crawled data and found many Weibo posts related to the Rio Olympics. This study speculates that the differences may be related to the Olympic Games held during that time period. At the time, Chinese athletes achieved excellent results in many competitions, which ignited the patriotic enthusiasm of the people and the pride of the nation. The mood of the whole network reached a peak at this time; thus, there were few negative Weibo posts. It can be seen that netizens’ emotional fluctuations are affected by multiple factors, such as air quality, seasons, and major events. The Pearson correlation coefficient is only a rough analysis. Next, this study will consider more factors through modeling in order to quantitatively study the relationship between the level of PM_2.5_ and the number of negative Weibo posts.

### 3.2. Interpreting the Estimation Results

#### 3.2.1. Test Results of the Correlation between PM_2.5_ Levels and People’s Emotional Intensity

The estimation results of Equation (1) are shown in Table 3. Model 1 and Model 2 use regression models to test the correlation between PM_2.5_ and the number of negative Weibo posts. Models 3 and 4 are used to test the correlation between PM_2.5_ and the share of negative Weibo posts.

We try to use the share of negative Weibo posts as the explained variable of the model. In Model 3, except for the two variables “precipitation” and “spring” that are not statistically significant, the other variables pass the significance test. The regression coefficient of each variable is small and the R^2^ of the model is not large (R^2^ = 0.1368), indicating that the variables are not sufficiently powerful to explain the share of negative Weibo posts. In Model 4, we add the variable “PM_2.5._” At this time, the three variables of “sea level pressure,” “spring,” and “autumn” all change from statistically significant to insignificant, and the coefficient of each variable is still small (C represents the regression coefficient of the variable; C_Sea level pressure_ = −0.0012, C_Spring_ = 0.0064, C_Autumn_ = −0.0146), and the R^2^ does not change much (R^2^ = 0.1509), indicating that the level of PM_2.5_ is not an influential variable on the share of negative Weibo posts. The goodness of fit of models 3 and 4 shows that the difference in the share of negative Weibo posts per day that the model can explain is less than 20%. Next, we examine other emotional indicators.

We use the number of negative Weibo posts as a measure of emotional intensity to verify the correlation between levels of PM_2.5_ and people’s emotional intensity. In Model 1, the variables “holiday,” “square of temperature,” “humidity,” “precipitation,” “sea level pressure,” and the seasonal variables are statistically significant. Among them, the coefficients of “holiday” and seasonal variables are both large and negative (C_Holiday_ = −22.1751, C_Spring_ = −46.7479, C_Summer_ = −78.2494, C_Autumn_ = −41.8634), indicating that they are influential variables and negatively correlated with the number of negative Weibo posts. During holidays, the public is free from the tension and anxiety of workdays, and their mood is relatively positive. Therefore, the number of negative Weibo posts can be expected to decrease significantly, consistent with the real-life mood of the public. For seasonal variables, we set winter as a collative variable. Given the depression that many people experience in winter, public sentiment is more positive in other seasons [39,46], so the number of negative Weibo posts is relatively small. In Model 2, after adding the variable “PM_2.5,_” the two variables of “temperature” and “wind speed” change from statistically insignificant to significant, while the variable “autumn” changes in reverse, indicating that the variable “PM_2.5_” and these three may interact with each other. In particular, the regression coefficient of the variable “summer” varies greatly from −78.2494 to −38.8843, indicating that the influence of “summer” on the number of negative Weibo posts decreases after adding the variable “PM_2.5._” The R^2^ of the model changes from 0.3343 to 0.5792, which is a big change, indicating that the level of PM_2.5_ has great significance for the interpretation of the number of negative Weibo posts. The results show that there is a significant positive correlation between PM_2.5_ levels and people’s emotional intensity (regression coefficient = 1.0168, *p* < 0.001), and the research hypothesis is established. We can see that there is an inverted U relationship between temperature and emotional intensity from the third column in Table 3, and the turning point is 12.3 °C, by our calculation. Based on our research in Beijing, we find that people prefer temperatures of about 54.1 °F. Based on the goodness of fit of different models, we select the number of negative Weibo posts as the final explained variable for the next analysis.

We conduct a heterogeneity test to verify the relationship between the level of PM_2.5_ and the number of negative Weibo posts in different dimensions. We group the full sample according to the season, whether the sample was taken on a holiday or on a weekend, and we estimate Equation (1) for each group.

#### 3.2.2. Regression Results Grouped by Season

Table 4 shows the test results of the relationship between the PM_2.5_ and the number of negative Weibo posts in the samples grouped by season. In model 1, only the variables “humidity,” “precipitation,” “sea level pressure,” and “PM_2.5_” passed the significance test, but the R^2^ of the model reached 0.7682, indicating that these variables have great significance for the interpretation of the number of negative Weibo posts in the spring samples. In Model 2, the variables “temperature,” “square of temperature,” “humidity,” “sea level pressure,” “wind speed,” and “PM_2.5_” are statistically significant. Among them, the regression coefficient of temperature is very large (C_Temperature_ = 8.04920), indicating that people’s emotions are more likely to fluctuate as the temperature increases in summer. However, the R^2^ of the model is slightly smaller (R^2^ = 0.3350), which indicates that these variables have little significance for the interpretation of the number of negative Weibo posts in summer samples. In model 3, the variables “holiday,” “temperature,” “square of temperature,” “precipitation,” ”wind speed,” and “PM_2.5_” are statistically significant. Among them, the coefficient of “holiday” is large (C_Holiday_ = −47.9146), indicating that holidays have a great influence on the number of negative Weibo posts in autumn samples. The R^2^ of the model is relatively large (R^2^ = 0.6598), which means that the above variables in the autumn sample have great significance for the interpretation of the number of negative Weibo posts. In model 4, only the three variables “weekend,” “square of temperature,” and “major event” do not pass the significance test, and the other variables are significant. Among them, the coefficient of “holiday” is large (C_Holiday_ = −67.8414). Holidays have a great impact on the number of negative Weibo posts in winter samples. This may be because, compared to other seasons, the holidays in the autumn and winter last longer, so people’s emotions can be better released. The model has a goodness of fit of 0.5936.

#### 3.2.3. Regression Results Grouped by Holiday and Weekend

The regression results grouped by holiday and weekend are shown in Table 5. In model 1, the variables “weekend,” “square of temperature,” “humidity,” “sea level pressure,” and “PM_2.5_” were statistically significant. The regression coefficient of “weekend” is large, indicating that the influence of weekends on the number of negative Weibo posts is great in holiday samples. The R^2^ of this model is large (R^2^ = 0.7013), indicating that the above variables have great significance for the interpretation of the number of negative Weibo posts in holiday samples. In model 2, “square of temperature,” “humidity,” “precipitation,” “sea level pressure,” “wind speed,” and “PM_2.5_” are statistically significant. The R^2^ of the model is large (R^2^ = 0.5668), indicating that the above variables have a good interpretation of the number of negative Weibo posts in non-holiday samples. In model 3, all variables are statistically significant. Among them, the coefficient of holiday is large (C_Holiday_ = −46.7156), which indicates that the impact of holidays on the number of negative Weibo posts is great in weekend samples, which also indicates that the festive atmosphere of holidays may be especially intense on weekends. The R^2^ of the model is also large (R^2^ = 0.5537), indicating that the above variables demonstrate a good interpretation of the number of negative Weibo posts in weekend samples. In model 4, the two variables “temperature” and “major event” are not significant, and the other variables pass the significance test. The coefficient of “holiday” is large (C_Holiday_ = −32.3766), indicating that the influence of holidays on the number of negative Weibo posts is great in non-weekend samples. The R^2^ of the model is large (R^2^ = 0.5628), indicating that the above variables have a great interpretation of the number of negative Weibo posts. Combined with Table 3, Table 4 and Table 5, major events are not statistically significant and do not have much impact on people’s emotional intensity.

From the results of Table 4 and Table 5, we can see that the variable “PM_2.5_” passes the significance test in samples divided by season, weekend, or holiday. Regression of the grouped samples does not affect the relationship between PM_2.5_ and people’s emotional intensity. The regression results show that there is still a significant positive correlation between PM_2.5_ levels and the number of negative Weibo posts. PM_2.5_ levels affect people’s emotional intensity, which further supports the research hypothesis.

## 4. Conclusions

Social media data can be used to provide a new perspective in research. In this study, we collected haze-related Weibo posts over two years by using crawling software and conducted a study using machine learning, correlation analysis, and linear regression model. We found that the level of PM_2.5_ is highly correlated with the emotional intensity of netizens.

The meaningful findings are as follows. (1) Taking the data over two years as an example, the Pearson correlation coefficient between the number of negative Weibo posts and PM_2.5_ levels reached 0.667. The posting behavior of netizens is basically consistent with the changing trend of PM_2.5_ levels and the time of occurrence of the most extreme points remains consistent, which shows that the level of PM_2.5_ has a significant influence on the posting behavior of netizens. (2) From the regression coefficient of the model, a 1 unit change in the level of PM_2.5_ causes a 1.0168 unit fluctuation in the number of negative Weibo posts, which verifies from a quantitative perspective that the emotional intensity of netizens is significantly and positively affected by PM_2.5_ levels. (3) In addition, during the modeling process, the variables for meteorological factors, seasons, and holidays passed the significance test, which shows that the public’s mood can be affected by multiple factors. (4) This study also recorded an interesting phenomenon: compared with other seasons, the correlation between PM_2.5_ levels and the number of negative Weibo posts in summer is not very high. The reason may be that the air quality is better in summer and emotional intensity is less affected by haze, which is a notable detail for future study.

We wish to pursue some further research in the future. First, we hope to see whether the conclusion is applicable in other areas. Second, we will use the location information in Weibo posts to research spatial heterogeneity of air pollution and Weibo’s negative posts. Third, we hope to build a dynamic prediction model for PM_2.5_ levels and to popularize the social media-based air quality monitoring method.

## Figures and Tables

**Table 1:** Correlation between PM_2.5_ levels and various statistical indicators (N = 113459).

Microblog Statistical Indicators	Pearson Correlation Coefficient	Statistical Significance
Number of positive microblogs	0.589 **	<0.01
Number of negative microblogs	0.667 **	<0.01
Total number of microblogs	0.625 **	<0.01
Positive microblogs’ share	−0.254 **	<0.01
Negative microblogs’ share	0.254 **	<0.01

Note: ** When the significance level (double test) is 0.01, the correlation is significant. Pearson correlation coefficient: (0.8, 1.) very strongly correlated; (0.6, 0.8) strongly correlated; (0.4, 0.6) moderately correlated; (0.2, 0.4) weakly correlated; (0, 0.2) very weakly correlated or not correlated.

**Table 2:** Correlation between PM_2.5_ levels and the number of negative microblogs (N = 113459).

Month	Pearson Correlation Coefficient	Statistical Significance
	In 2016	
January	0.752 **	<0.01
February	0.856 **	<0.01
March	0.930 **	<0.01
April	0.689 **	<0.01
May	0.732 **	<0.01
June	0.602 **	<0.01
July	0.626 **	<0.01
August	0.461 **	<0.01
September	0.734 **	<0.01
October	0.813 **	<0.01
November	0.711 **	<0.01
December	0.771 **	<0.01
	**In 2017**	
January	0.616 **	<0.01
February	0.887 **	<0.01
March	0.829 **	<0.01
April	0.841 **	<0.01
May	0.927 **	<0.01
June	0.527 **	<0.01
July	0.566 **	<0.01
August	0.610 **	<0.01
September	0.804 **	<0.01
October	0.846 **	<0.01
November	0.699 **	<0.01
December	0.823 **	<0.01

Note: ** When the significance level (double test) is 0.01, the correlation is significant.

**Table 3:** Test results of correlation between PM_2.5_ levels and people’s emotional intensity.

Explanatory Variable	Explained Variable			
	Number of Negative Weibo Posts		Share of Negative Weibo Posts	
	Model 1	Model 2	Model 3	Model 4
Weekend	5.8296	3.8199	−0.0289 ***	−0.0294 ***
	(6.5029)	(5.2182)	(0.0071)	(0.0071)
Holiday	−22.1751 **	−44.2820 ***	−0.0270 **	−0.0323 ***
	(9.1351)	(9.0503)	(0.0109)	(0.0111)
Temperature	0.1088	3.3306 ***	−0.0046 ***	−0.0038 ***
	(1.1620)	(1.0039)	(0.0011)	(0.0011)
Square of temperature	−0.1203 ***	−0.1350 ***	0.0001 ***	0.0001 ***
	(0.0327)	(0.0268)	(0.0000)	(0.0000)
Humidity	2.3040 ***	0.5547 ***	0.0009 ***	0.0005 **
	(0.2952)	(0.1722)	(0.0002)	(0.0002)
Precipitation	−1.5675 ***	−0.2797 *	−0.0002	0.0001
	(0.5098)	(0.1575)	(0.0006)	(0.0006)
Sea level pressure	−1.2848 **	1.0594 *	−0.0018 **	−0.0012
	(0.6455)	(0.5485)	(0.0007)	(0.0007)
Wind speed	0.0508	2.7047 ***	−0.0033 ***	−0.0027 ***
	(0.6709)	(0.6352)	(0.0007)	(0.0007)
Major event	−0.2190	0.7872	−0.0150 **	−0.0148 **
	(6.5950)	(5.2985)	(0.0076)	(0.0074)
Spring	−46.7479 ***	−58.0878 ***	0.0091	0.0064
	(12.5696)	(10.9529)	(0.0111)	(0.0109)
Summer	−78.2494 ***	−38.8843 ***	−0.0422 **	−0.0327 *
	(15.2589)	(11.2806)	(0.0175)	(0.0172)
Autumn	−41.8634 ***	−11.4606	−0.0220 **	−0.0146
	(14.8600)	(11.0593)	(0.0093)	(0.0094)
PM2.5		1.0168 ***		0.0002 ***
		(0.0935)		(0.0000)
R^2^	0.3343	0.5792	0.1368	0.1509

Note: The values in the table represent the correlation coefficients of the variables of the regression model, and the standard error of each coefficient is in parentheses. ***, **, * indicate significance at the levels of 0.01, 0.05 and 0.1, respectively. Winter is the default variable.

**Table 4:** Regression results grouped by season.

Explanatory variable	Explained Variable: Number of Negative Weibo Posts			
	Spring Sample	Summer Sample	Autumn Sample	Winter Sample
	Model 1	Model 2	Model 3	Model 4
Weekend	−2.2482	0.0871	6.2589	−8.4189
	(2.6921)	(2.1938)	(11.2871)	(13.5364)
Holiday	−5.6358	−1.2324	−47.9146 ***	−67.8414 ***
	(3.6301)	(3.0269)	(13.7614)	(20.1369)
Temperature	0.6360	8.0492 **	7.9258 ***	5.6999 **
	(1.0657)	(3.3116)	(2.2704)	(2.3145)
Square of temperature	−0.0424	−0.1510 **	−0.3072 ***	−0.2588
	(0.0296)	(0.0656)	(0.0700)	(0.2622)
Humidity	−0.1998 *	0.1838 ***	−0.3889	2.5306 ***
	(0.1091)	(0.0656)	(0.4263)	(0.7840)
Precipitation	0.4642 *	−0.0656	1.9974 *	−20.4136 ***
	(0.2790)	(0.0478)	(1.0437)	(7.0949)
Sea level pressure	0.6597 **	0.4025 **	1.2726	2.8981 **
	(0.3241)	(0.2010)	(1.2203)	(1.3610)
Wind speed	0.4996	1.2986 **	5.2688 ***	4.3613 ***
	(0.3360)	(0.6216)	(1.2799)	(1.2222)
Major event	0.6764	−0.4948	6.8629	1.5558
	(4.9188)	(1.7036)	(11.7907)	(18.1195)
PM2.5	0.5568 ***	0.1989 ***	1.8648 ***	0.8980 ***
	(0.0430)	(0.0558)	(0.2196)	(0.1695)
R^2^	0.7682	0.3350	0.6598	0.5936

Note: The values in the table represent the correlation coefficients of the variables of the regression model, and the standard error of each coefficient is in parentheses. ***, **, * indicate significance at the levels of 0.01, 0.05 and 0.1, respectively. Winter is the default variable.

**Table 5:** Regression results are grouped by holiday and weekend.

Explanatory variable	Explained Variable: Number of Negative Weibo Posts			
	Holiday Sample	Non-Holiday Sample	Weekend Sample	Non-Weekend Sample
	Model 1	Model 2	Model 3	Model 4
Weekend	18.0618 *	5.5692		
	(10.7563)	(5.6416)		
Holiday			−46.7156 ***	−32.3766 ***
			(17.3686)	(8.4650)
Temperature	−0.6179	0.4666	3.5571 **	−0.2817
	(1.3368)	(0.8598)	(1.5658)	(0.9612)
Square of temperature	0.1149 **	−0.0763 ***	−0.1586 ***	−0.0434 *
	(0.0501)	(0.0212)	(0.0429)	(0.0238)
Humidity	1.0464 ***	1.1335 ***	1.3154 ***	1.0190 ***
	(0.2814)	(0.1753)	(0.3307)	(0.1747)
Precipitation	−2.7737	−0.5381 ***	−1.1821 **	−0.4681 **
	(1.8766)	(0.2042)	(0.5630)	(0.1972)
Sea level pressure	3.5770 ***	1.3219 **	2.1149 *	1.4178 **
	(0.9727)	(0.5838)	(1.2398)	(0.6250)
Wind speed	0.2875	3.2390 ***	3.7215 ***	2.7884 ***
	(0.9436)	(0.7243)	(1.2767)	(0.7722)
Major event	−25.5973	−3.0546	−12.4966 *	3.7993
	(15.7975)	(5.7231)	(7.1763)	(6.9226)
PM2.5	0.5046 ***	1.0451 ***	0.9978 ***	0.9540 ***
	(0.0983)	(0.0978)	(0.1704)	(0.1075)
R^2^	0.7013	0.5668	0.5537	0.5628

Note: The blank is the corresponding variable that is rejected because of the differences in the samples. The values in the table represent the correlation coefficients of the variables of the regres-sion model, and the standard error of each coefficient is in parentheses. ***, **, * indicate signifi-cance at the levels of 0.01, 0.05 and 0.1, respectively. Winter is the default variable.

## Data Availability

The air quality data can be found on the China National Environmental Monitoring Centre website (http://www.cnemc.cn (accessed on 19 May 2021)). The temperature, humidity, visibility, and wind speed data can be found on the Wunder-ground platform (https://www.wunderground.com (accessed on 19 May 2021)).
